# Tumescent anaesthesia for post burn contracture release

**DOI:** 10.4103/0019-5049.72656

**Published:** 2010

**Authors:** Rajeev Sharma

**Affiliations:** Department of Anaesthesia, ESI Hospital, Rohini, New Delhi, India

Sir,

I read with interest the article titled “Role of ketamine in fiberoptic era” by Chand *et al*.[1] I congratulate the authors for successful airway management of a case of post-burn contracture presenting with fixed flexed neck deformity. The authors used intravenous ketamine along with lidocaine 2% for the release of neck contracture before an LMA (Laryngeal Mask Airway) was placed for ventilation.

According to the authors, release of contracture under local anaesthesia could not be done fearing that the safe dose of local anaesthetic would have been exceeded. I do not agree to them on this point. “Tumescent anaesthesia” is a technique for delivery of local anaesthesia that maximises safety by using pharmacokinetic principles to achieve extensive regional anaesthesia of skin and subcutaneous tissue. The subcutaneous infiltrations of a large volume of very dilute lidocaine (as low as 0.1%) and epinephrine causes the targeted tissue to become swollen and firm, or tumescent, and permits procedures to be performed on patients without subjecting them to the inherent risks of local anaesthetic toxicity. The use of diluted lidocaine allows administration of doses upto 35–55 mg/kg. This technique has been used safely for procedures like harvesting skin grafts,[2] liposuction and post burn neck contracture release.[3]
